# Inherited desmoplastic trichoepitheliomas

**DOI:** 10.1111/ced.13876

**Published:** 2019-01-29

**Authors:** M.‐L. Lovgren, N. Rajan, S. Joss, L. Melly, M. Porter

**Affiliations:** ^1^ Dermatology Department West Glasgow Ambulatory Care Hospital NHS Greater Glasgow and Clyde Dalnair Street Yorkhill Glasgow G3 8SJ UK; ^2^ Institute of Genetic Medicine Newcastle University Newcastle upon Tyne UK; ^3^ Genetics Department Queen Elizabeth University Hospital NHS Greater Glasgow and Clyde Glasgow UK; ^4^ Histopathology Department Queen Elizabeth University Hospital NHS Greater Glasgow and Clyde Glasgow UK

Desmoplastic trichoepitheliomas (DTE) are rare adnexal tumours arising on sun‐exposed sites. Familial DTEs have rarely been reported. We describe two sisters with multiple early‐onset DTEs, and a basal cell carcinoma (BCC), suggesting an underlying genetic predisposition.

**Figure 1:**
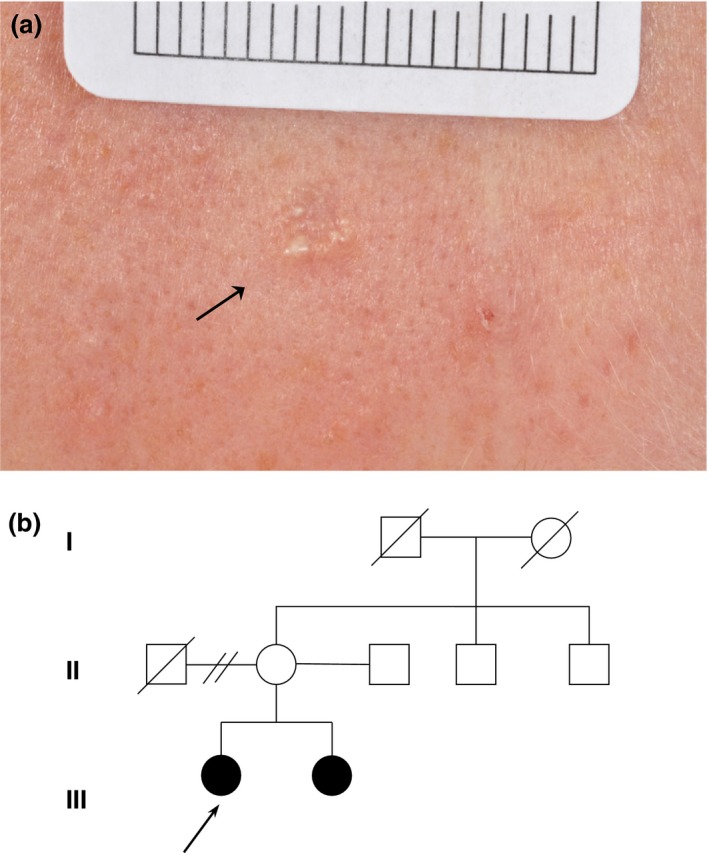
(a) The proband had a central forehead lesion (black arrow) demonstrating an ill‐defined skin‐coloured lesion 3 mm in size, with milia‐like cysts. (b) Pedigree demonstrating a family history of desmoplastic trichoepitheliomas and a basal cell carcinoma; black arrow indicates the proband.

**Figure 2:**
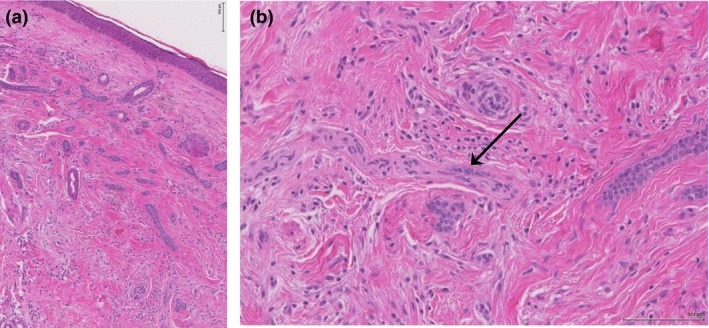
(a) Incisional biopsy from the proband's sister's right mandible demonstrating small keratocysts and thin strands of basaloid cells in the superficial dermis with a single focus of perineural invasion and no subcutis invasion. Immunocytochemistry for CK20, epidermal membrane antigen and carcinoembryonic antigen was negative. No retraction artefact or peripheral palisading was seen, and BerEP4 was only focally positive. (b) Perineural invasion in the right mandibular lesion (black arrow). Haematoxylin and eosin, original magnification (a) × 100; (b) × 200.

A 19‐year‐old woman underwent excision of a central forehead lesion (Fig. 1a) and a similar right cheek lesion, which were both histologically consistent with DTEs. Her 30‐year‐old half‐sister (Fig. 1b) had a nodular BCC and a DTE excised from the left and central forehead, respectively. At the age of 31 years, she then developed an ill‐defined right mandibular skin‐coloured plaque. Incisional biopsies suggested DTE or microcystic adnexal carcinoma (MAC), but DTE with perineural invasion (PNI) was confirmed on excision (Fig. 2a,b). The sisters’ mutual mother had no similar skin lesions on examination. Genetic testing of coding exons of the *CYLD* gene on lymphocyte DNA taken from the older sister was negative.

Trichoepitheliomas (TEs) are benign hair follicle tumours, divided into single TEs, multiple TEs and DTEs. The latter presents in young and middle‐aged women, as slow‐growing skin‐coloured, facial annular plaques or nodules with a depressed centre. By contrast, MACs are rare locally aggressive cutaneous cancers, uncommonly metastasizing, presenting as indolent, skin‐coloured or yellowish facial lesions. DTEs and MACs are difficult to distinguish clinically and histologically with small biopsies, so deep biopsies are required. Both demonstrate basaloid cords and nests within a fibrous stroma. DTEs are well‐circumscribed and confined to the dermis, whereas MACs are more diffuse and invade the subcutis with PNI in 80% of cases.^1^ No single immunohistochemical stain distinguishes between them. The skin tumours of both sisters were clinically subtle, and histologically all, except the nodular BCC, resembled DTEs. The mandibular DTE in the proband's sister demonstrated PNI and a diffuse growth pattern. Jedrych *et al*. reported seven histologically classic DTEs with PNI.^1^ Five cases were followed‐up (2 months to 4 years) with no recurrence; the authors concluded DTEs with PNI can be managed conservatively, whereas others suggest surgical excision or Mohs micrographic surgery.^2^

Three families with DTEs have been reported previously.^3^ A 57‐year‐old woman had five DTEs and a BCC, while her son had two DTEs and her mother had a cylindroma, suggesting autosomal dominant inheritance.^4^ Familial DTEs may be under‐reported given the benign nature and subtle clinical appearances.^3^ Familial MACs have also been reported in two siblings, one of whom had a BCC and a papillary thyroid carcinoma.^5^ Genetic analysis was not performed in these cases. Given the report of cylindroma in a family with DTE^4^ and the strong association of cylindroma and mutations in the *CYLD* gene, we carried out sequencing of *CYLD* in the older sister. Our report of an absence of *CYLD* mutation in this case is an important negative finding, suggesting that alternative genes may play a role in the pathogenesis of DTEs.

Regular follow‐up and low threshold for biopsies led to early tumour diagnosis in our pedigree, minimizing the cosmetic and functional impact. Adequate follow‐up of larger series of DTEs with PNI is required to establish their behaviour. Our cases raise questions about the genetic and histopathological relationship between DTEs, MACs and BCCs.
